# DNA-Based Biosensors for the Biochemical Analysis: A Review

**DOI:** 10.3390/bios12030183

**Published:** 2022-03-20

**Authors:** Yu Hua, Jiaming Ma, Dachao Li, Ridong Wang

**Affiliations:** State Key Laboratory of Precision Measuring Technology and Instruments, Tianjin University, Tianjin 300072, China; huayu_19@tju.edu.cn (Y.H.); majiaming@tju.edu.cn (J.M.); dchli@tju.edu.cn (D.L.)

**Keywords:** DNA-based biosensors, biomedical analysis, DNA aptamer, DNAzyme, hybridization chain reaction, catalytic hairpin assembly, DNA framework, DNA origami, DNA nanotechnology

## Abstract

In recent years, DNA-based biosensors have shown great potential as the candidate of the next generation biomedical detection device due to their robust chemical properties and customizable biosensing functions. Compared with the conventional biosensors, the DNA-based biosensors have advantages such as wider detection targets, more durable lifetime, and lower production cost. Additionally, the ingenious DNA structures can control the signal conduction near the biosensor surface, which could significantly improve the performance of biosensors. In order to show a big picture of the DNA biosensor’s advantages, this article reviews the background knowledge and recent advances of DNA-based biosensors, including the functional DNA strands-based biosensors, DNA hybridization-based biosensors, and DNA templated biosensors. Then, the challenges and future directions of DNA-based biosensors are discussed and proposed.

## 1. Introduction

Due to superior biocompatibility [1], thermal stability [2,3,4,5], and alternative functionalization [6,7,8], deoxyribonucleic acid (DNA) is becoming a fascinating biological material used for biosensing. It is widely acknowledged that DNA and its assembly structure can be applied for detecting specific targets, including nucleic acids, proteins, metal ions, and small biological molecules [9,10,11,12,13,14,15]. With the development of DNA nanotechnology, dynamic networks based on DNA hybridization can be used to amplify the signals of biosensors. In addition, DNA is also a powerful material to assemble complex 3D nanostructures and organize the other functional units.

Compared to commonly used bioprobes, more durable biological activity, remarkable addressability, and adjustable rigidity make DNA a promising candidate for intelligent biosensing. It has been reported that through manual screening and modification, DNA probes, like aptamer, have better thermal stability [16,17,18], adjustable biological affinity [19,20,21], and higher resistance to nucleases enzyme attack [22,23]. DNA can also be used to build programmable supermolecule structures as the template to realize the precise controlling of the spatial position of the modifications, which could significantly improve the performance of the biosensor and even inspire researchers to propose novel biosensors [24,25,26].

Based on the great potential of DNA biosensors. A large number of reports have reviewed the basic principles and the recent advances of the biosensors based on DNA aptamers [27,28,29,30,31], DNAzyme [32,33,34,35], DNA hairpins [36,37,38,39], DNA tiles [40,41,42], and DNA origami [43,44,45,46,47]. Although these reports have introduced DNA biosensors from different aspects in detail, a comprehensive overview of DNA-based biosensors is still needed.

**Figure 1:**
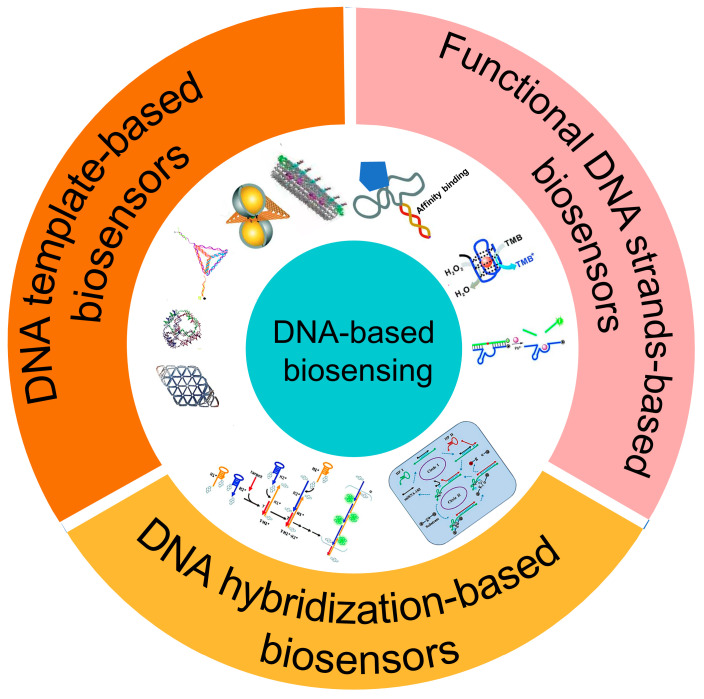
Schematic diagram of different DNA-based biosensors.

This review summarizes the state-of-the-art research progress for different types of DNA-based biosensors (as shown in Figure 1), including functional DNA strand-based biosensors, DNA hybridization-based biosensors, and DNA template-based biosensors. Functional DNA strand-based biosensors refer to biosensors that use functional DNA strands to recognize certain targets, like aptamer biosensors [48] and DNAzyme biosensors [49]. DNA hybridization-based biosensors refer to biosensors that use enzyme-free nucleic acid amplification strategies to enhance their responses, like the biosensors based on the DNA hairpin [37], the hybridization chain reaction (HCR) [50], and catalytic hairpin assembly (CHA) [51]. DNA template-based biosensors mean that the biosensors are decorated with a DNA template (the supermolecule DNA assembly structures with programmable anchoring points), like the biosensors based on DNA tetrahedron [52] and DNA origami [53]. The basic principle and the application of these biosensors are introduced. Moreover, we summarize the characters and applications based on functional DNA strands, DNA hybridization, and DNA template in biosensing. Then, potentials, challenges, and development trends of DNA-based biosensors are given.

## 2. Functional DNA Strands-Based Biosensors

Biosensors rely on the interaction of a molecular probe with a specific affinity to the target analyte [54]. In the past few decades, enzymes [55], antibodies [56,57], and oligonucleotides [58,59] have been widely used as biosensor probes with specific recognition functions. Compared with the biosensors based on an enzyme or antibody, biosensors based on DNA probes have advantages of high thermal tolerance, easy modification, and efficient surface regeneration because of the stable chemistry [60]. More significantly, DNA probes with different affinity for target analytes can be obtained through directed screening of DNA libraries [61,62]. In this section, biosensors based on two kinds of functional DNA strands, DNA aptamer, and DNAzyme are introduced.

### 2.1. DNA Aptamer Biosensors

Aptamer refers to a series of synthetic nucleic acids capable of binding to a specific target. The first aptamer was obtained in 1990 by Ellington et al. [63]. Compared with the traditional bioprobes like the antibody, DNA aptamers could better adapt to extremely high temperatures, pH values, and high ionic concentrations. Furthermore, a DNA aptamer allows much simpler modification of functional groups without losing biological activity. Additionally, the manufacturing cost is also dramatically reduced with the rapid development of DNA synthesis technology. All these advantages facilitate the wide application of DNA aptamers in various biosensors.

**Figure 2:**
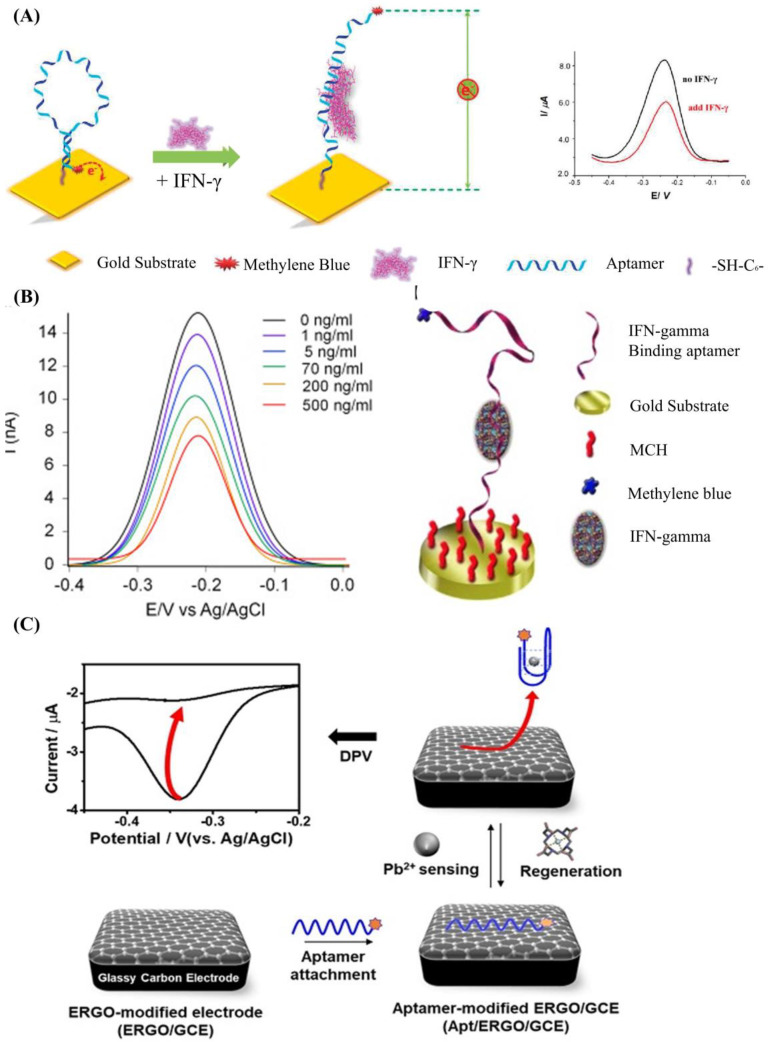
(**A**) Schematic of aptamer-based electrochemical sensor for IFN-γ. Reprinted (adapted) with permission from [64]. Copyright (2010) American Chemical Society. (**B**) Demonstration of the MB-tagged aptamer modified electrode. Reprinted (adapted) with permission from [65]. Copyright (2014) Elsevier. (**C**) Schematic diagram depicting the fabrication of Ap/ERGO/GCE-based electrochemical aptasensor for the detection of Pb^2+^. Reprinted (adapted) from [77]. Copyright (2019) MDPI, Basel, Switzerland, under the Creative Commons Attribution License.

The most used strategy to detect a biotarget with a DNA aptamer is to functionalize the DNA aptamer with a report molecule (ferrocene, methylene blue) and an immobilization molecule (alkane thiol, alkane amino, streptavidin, and hydrazoate) at the 5′ end and 3′ end of the DNA strand, respectively. The change of DNA aptamer construction could be read out by detecting the electrochemical change on the electrode surface. Liu et al. developed an electrochemical sensor based on a 34-mer IFN-γ-binding aptamer for interferon-gamma (IFN-γ) detection [64] (Figure 2A). In their study, the proposed DNA aptamer was fixed on the surface of the electrode through a covalent reaction of gold and alkyl mercaptan. When there was no specific target, the DNA aptamer self-folded to form a secondary spatial loop structure, which caused the reporting molecules to contact the sensor electrode surface. The combination of the target and the DNA aptamer changed the aptamer’s conformation, enlarging the distance between the reporting molecule and the electrode surface. This led to the change of electronic transfer efficiency between the reporting molecules and the electrode surfaces. The limit of detection (LOD) reached 0.06 nM, and the linear detection range was extended to 10 nM. In a similar study (Figure 2B), Chen et al. developed an electrochemical sensor array based on the 34-mer IFN-γ binding aptamer modified with MB and disulfide, using standard semiconductor processes [65]. This sensor could detect IFN-γ ranging from 1 to 500 ng/mL with a LOD of 1.3 ng/mL.

A DNA aptamer can also be decorated with conjugated polymers, which has been widely applied as the report tags in fluorescent and colorimetric biosensors because of its excellent optoelectrical properties [66]. The DNA aptamer can absorb onto the conjugated polymers through the electrostatic force [67]. When binding with the biotarget, the conformation change of the DNA aptamer will lead to the adjustment of the conjugated polymer’s constructure, which will influence the absorption and emission wavelength of the conjugated polymers [68]. This method has been successfully used to detect human α-thrombin, with a LOD of 2 × 10^−15^ M [69].

Although functional DNA aptamer biosensors exhibit many advantages, a complex modification process is needed to immobilize the aptamers onto the sensor surface firmly. Graphene oxide (GO), which shows excellent photoelectric properties and carrier transport capability, is a widely used 2D material to simplify the process [70,71,72,73,74,75]. It has been reported that single DNA strands could be tightly adsorbed on the GO surface through base-stacking and hydrogen bonding without chemical modification [76]. Yu et al. proposed a simple method to detect microscale Pb^2+^ using electrochemical aptamer sensors modified with electrochemically reduced graphene oxide (ERGO) [77]. In their work (shown in Figure 2C), the ERGO was deposited on the glassy carbon electrode, and the guanine-rich DNA aptamer modified with methylene blue (MB-aptamer) was physically adsorbed onto the ERGO through the π-π interaction. The Pb^2+^ led the MB-aptamer to fold to G-quadruplex and separate from the electrode surface, which could weaken the electrochemical signal of the sensor surface. This strategy had a linear range from 10^−5^ to 10^−9^ M and a LOD of 0.51 fM.

**Figure 3:**
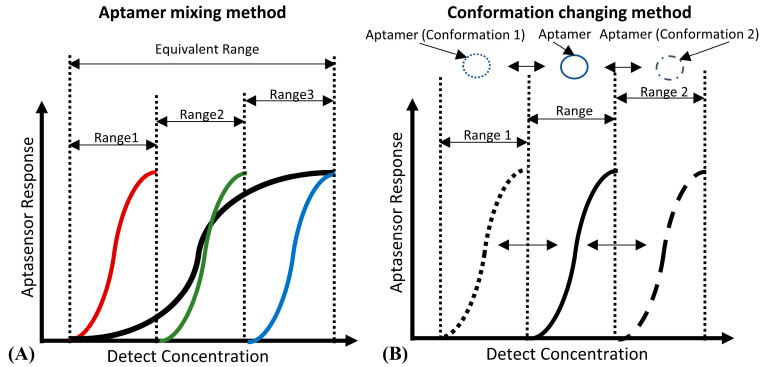
(**A**) The principle of the “aptamer mixing method”. It can be seen that the detection ranges of aptamers, which have a high affinity (marked in red), medium affinity (marked in green) and low affinity (marked in blue) to the same biotarget, are narrow. However, the equivalent detection range of their mixture is wide. (**B**) The principle of the “conformation changing method”. By inducing the aptamer to change to the conformation with high affinity (Conformation 1), and the conformation with low affinity (Conformation 2), the detection range of the aptamer can shift to the low detection concentration area (Range 1) and the high detection concentration area (Range 2) respectively.

For DNA aptamer biosensors, the dynamic range and sensitivity, which are limited by the Langmuir isothermal adsorption model, are also not flexible enough to suit the different ranges of detection concentration required [78,79]. As shown in Figure 3A, aptamers with different detection ranges can be mixed to achieve a wider detection range. This method is quite suitable for the detection of several viruses [80] and inflammatory biomarkers, the detection ranges of which may span several orders of magnitude [81]. However, due to the unchanged maximum response of the sensor, the sensitivity of this kind of biosensor will be decreased. Figure 3B shows that the detection range can also be changed by adjusting the aptamers’ conformation, which shifts the detection range of the aptamer rather than expands it. Therefore, its biosensing sensitivity will not be decreased, which is suitable for applications like the detection of cancer biomarkers [82] (high sensitivity instead of a wide detection range is preferred).

In addition to improving the biosensing performance, the cost of these biosensors should be taken into consideration, which indicates that it is meaningful to develop reusable biosensors. One commonly used way is to flush the sensor surface with running buffer rapidly for a long time to recover the sensor surface. A prominent drawback is the baseline of the sensor may drift after flushing the sensor surface, which can influence the detection range of the sensor. Moreover, the overly fast flow will physically damage the modification of the sensor surface, especially when the aptamers are immobilized onto the sensor through electrostatic force. Another way to recover the sensor surface is to use a denaturant, a chaotropic, or surfactants. However, these reagents are harmful to the bioactivity of the sensor. To overcome the above-mentioned disadvantages, a method called DNA aptamer substitution was proposed to realize the recovery of the sensor surface more effectively and more simply [83]. Compared to the two methods above, there was no residual biotarget on the sensor surface, and this recovery process did not change the surface distribution of the DNA aptamer.

### 2.2. DNAzyme Biosensors

Enzymes, which have high catalytic efficiency, are widely used in biosensing [84,85,86,87]. However, the enzymatic activity can be affected by many environmental factors, which limit the application in biosensors [88,89]. It is found that some manually screened DNA strands which are more adaptive to the environment and resistive to the nuclease degradation also have enzymatic activity [32,90]. These DNA strands, named DNAzyme, have excellent potential to be efficient biometric probes. Among the DNAzymes with different functions, the nucleic acid cleavage function and the catalytic function of peroxides are widely used in biosensing.

**Figure 4:**
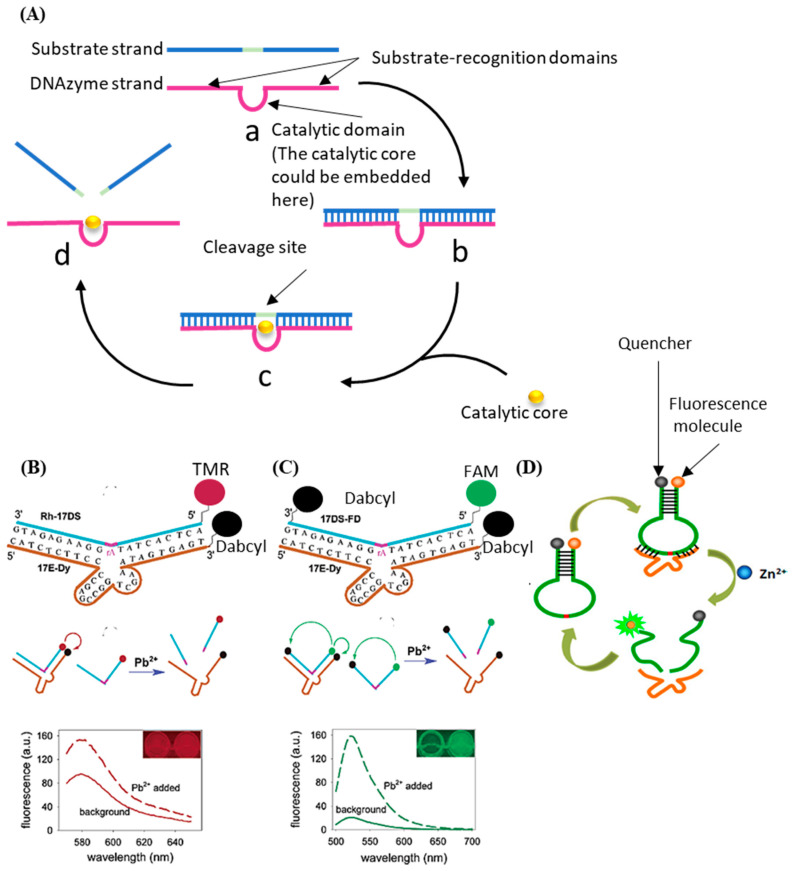
(**A**) The basic principle of the DNAzyme with nucleic acid cleavage function. (**B**) Schematic diagram of previous DNAzyme-based Pb^2+^ sensor. (**C**) Schematic diagram of improved DNAzyme-based Pb^2+^ sensor. Reprinted (adapted) with permission from [103]. Copyright (2003) American Chemical Society. (**D**) Schematic diagram of DNAzyme-based amplified biosensor. Reprinted (adapted) with permission from [104]. Copyright (2013) American Chemical Society.

The DNAzyme with nucleic acid cleavage function consists of a loop-shaped catalytic domain flanked by two substrate-recognition domains [91]. Figure 4A shows the basic principle of DNAzyme with nucleic acid cleavage function. The substrate-recognition domains can capture the substrate strand through the Watson–Crick model. The cleavage will not be activated without the existence of the catalytic core. When the external catalytic core embeds into the catalytic domain of the DNAzyme, the cleavage process is stimulated. The substrate strand is then cut off and separated from the substrate-recognition domains because the melting temperature decreases [92,93,94,95,96]. During this process, only certain small molecules (like metal ions [97,98,99] and amino acids [100]) can serve as the catalytic core of the corresponding DNAzyme. Therefore, the stimulation of the cleavage process is highly specific, which can be utilized for small-molecule detection.

The most commonly used method based on this principle is called the molecular beacon [101]. Generally, there are three types of molecule beacons: the beacon with a single quencher, the beacon with double quenchers, and the beacon with the hairpin-shaped substrate (shown in Figure 4B–D, respectively). The beacon with a single quencher refers to the molecule beacon modified with one fluorescence molecule and one quencher. Li et al. developed a highly sensitive DNAzyme biosensor based on the beacon with a single quencher to detect lead ions [102]. In this study, the fluorescence sensor was constructed by modifying the 5′ end of the substrate DNA (Rh-17DS) with fluorophore 6-carboxylic tetramethylrhodamine (TMR) and modifying the 3′ end of the DNAzyme chain (17E-Dy) with fluorescence quencher 4-(4′-dimethylaminophenylazo) benzoic acid (Dabcyl). The Pb^2+^ activated the DNAzyme’s cleavage process, leading to the separation of the fluorescence molecule (TMR) and the quencher (Dabcyl). Therefore, the FRET between the TMR and the Dabcyl was interrupted, and the intensity of the fluorescence was enhanced. Their proposed sensor had an 80-times-higher response for Pb^2+^ than for the other divalent metal ions. However, this biosensor only worked well at low temperatures. When the ambient temperature exceeded the melting temperature, some of the DNA enzymes would release the substrate strand without cleaving, which would cause high background noise and weaken the selectivity of the method. To solve this issue, the beacon with double quenchers, which refers to the beacon modified with one fluorescence molecule and double quenchers, was proposed. Liu et al. proposed an improved fluorescent DNAzyme sensor based on the beacon with double quenchers [103]. As shown in Figure 4C, the fluorescent molecule (FAM) was quenched not only by the quencher (Dabcyl) on the DNAzyme (17E-Dy) but also by the quencher (Dabcyl) on the substrate strand (Rh-17DS). Even when the substrate strand was separated from the DNAzyme abnormally, this clever design could still keep the fluorescent molecules in a quenching state, because the Dabcyl on the substrate strand could still quench the FAM. Their results showed that the proposed sensor’s relative fluorescence intensity was increased by 60%. The beacon with hairpin-shaped substrate refers to the stem-loop of the DNA hairpin that serves as the substrate of the DNAzyme. Zhao et al. developed a DNAzyme-based amplified biosensor [104]. As shown in Figure 4D, the fluorescent-quench pair was modified at the DNA hairpin’s end. The cleavage site was set on the loop of the hairpin. Induced by zinc ions, the DNAzyme cut the loop and released fragments of nucleic acid with fluorescent-quenching molecules to increase the intensity of the fluorescence. This strategy did not need to modify DNAzyme, avoiding the decrease of the DNAzyme’s catalytic activity.

**Figure 5:**
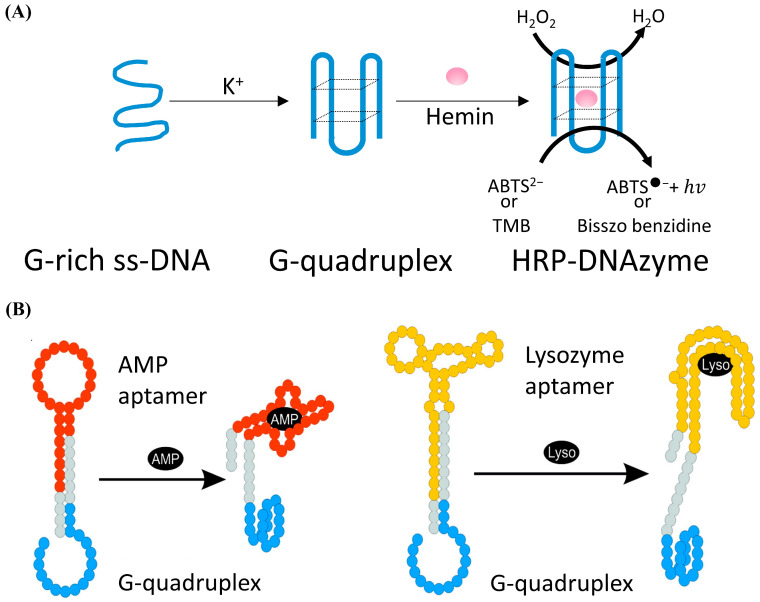
(**A**) Schematic of the form and the function of the HRP-DNAzyme. (**B**) Schematic analysis of adenosine monophosphate (AMP) or lysozyme (Lyso) by the aptamer—DNAzyme hairpin structure; reprinted (adapted) with permission from [106]. Copyright (2009) American Chemical Society.

DNAzyme with the catalytic activity of peroxides was firstly discovered by Sen et al. in 1998 [105]. As shown in Figure 5A, in the presence of potassium ions, DNA sequences rich in guanine can combine with hemin to form HRP-DNAzyme, which could catalyze 2,2′-azino-bis (3-ethylbenzothiazoline-6-sulfonic acid) (ABTS^2−^) or tetramethylbenzidine (TMB) to the colored products. HRP-DNAzymes are commonly applied as report tags of DNAzyme and DNA aptamer biosensors because HRP-DNAzyme can produce intense fluorescent or colorimetric signal and will not largely affect the bioactivity of these DNA biosensors. Willner et al. designed an aptamer-HRP-DNAzyme hairpin biosensing structure for detecting AMP and lysozyme [106]. In their research (shown in Figure 5B), the hybridization of the analyte with the DNA aptamer sequence causes the DNAzyme to untie from the stem and form a G-quadruplex structure. Catalyzing the ABTS^2−^ to the colored ABTS^●^^−^ by the HRP-DNAzyme, an amplified optical signal could be obtained at the same time for detecting respective analytes. HRP-DNAzyme can also catalyze the polymerization of aniline to form polyaniline, which shows superior SPR signal-enhancing ability. Based on this, Li et al. proposed an SPR biosensor based on HRP-DNAzyme for signal amplification [107]. In the study, bleomycin was used as the target, and a much lower LOD down to 0.35 pM was realized.

## 3. DNA Hybridization-Based Biosensors

Rapid and sensitive detection of specific biomarkers is of great importance in biochemical analysis, especially in the global pandemic of COVID-19 nowadays [108,109,110]. At present, the widely used nucleic acid detection methods include polymerase chain reaction (PCR) [111,112], loop-mediated thermal amplification (LAMP) [113], DNA chip-based microarray [114], and enzyme-linked immunosorbent assay (ELISA) [115]. However, these methods require complex testing equipment, professionally trained inspectors, long incubating time, and complex manufacturing processes. These shortcomings limit the application of these methods in airports, train stations, communities, etc. The detection technique based on DNA hybridization amplification, which is of high detection speed, sensitivity, and stability, shows great potential to overcome the above-mentioned drawbacks. In this session, nucleic acid hybridization-based biosensors, including the DNA hairpin biosensors, the HCR biosensors, and the CHA biosensors used to detect specific biology targets, were introduced.

### 3.1. Biosensor Probes Based on DNA Hairpin

DNA hairpin refers to the hairpin structure formed by ssDNA with a self-complementary sequence. Based on Watson-Crick’s pairing principle, this structure shows high specificity and can convert the hybridization to the physical signal easily. Thus, the DNA hairpin-based probe can be a powerful tool for detecting target nucleic acid fragments. Fang et al. proposed a kind of molecular beacon for surface-immobilized DNA hybridization studies [116]. In their research, the hairpin was modified with tetramethylrhodamine (TMR) and dimethylaminoazobenzen aminoexal-3-acryinido (DBCAL) as fluorescence–quenching pairs. TMR and DBCAL were detached with the opening of the hairpin by the target, which led to the increase of the fluorescence signal. This sensitivity could reach as low as the nanomolar scale. Fan et al. developed an electrochemical DNA (E-DNA) biosensor based on the DNA hairpin to detect the sequence-specific DNA [117]. In the study, a DNA hairpin, which was modified with methylene blue (MB) and a ferrocene molecule, was immobilized onto the working electrode through the covalent reaction between gold and hydro sulphonyl. As the sequence-specific DNA opened the hairpin and caused the detachment of MB from the sensor surface, the electrochemical signal of the sensor decreased. The LOD of this sensor could reach as low as 10 pM. Compared with the fluorescence method, this biosensor did not need bulky equipment and was not affected by the sample’s light transmittance. However, the response current was inversely proportional to the target molecule’s concentration, which indicated that the sensor response under high concentration might be drowned in background noise. One method to overcome the drawback is designing a hairpin structure based on “signal gain mode” [118]. Another method is introducing a reference probe to reduce the background noise [119].

### 3.2. Signal-Enhanced Biosensors Based on DNA Hybridization

Although the biosensors based on DNA probes like aptamers, DNAzymes, or DNA hairpins have achieved high sensitivity, chemical stability, and low manufacturing costs, the LOD is still limited by the surface density of DNA probes. Denser surface distribution of DNA biometric elements could theoretically increase the probability of combining the target and sensor surface probe [120]. However, the steric hindrance effect caused by the over-density of DNA probes on the sensor surface prevents the target from binding to the immobilized probes.

**Figure 6:**
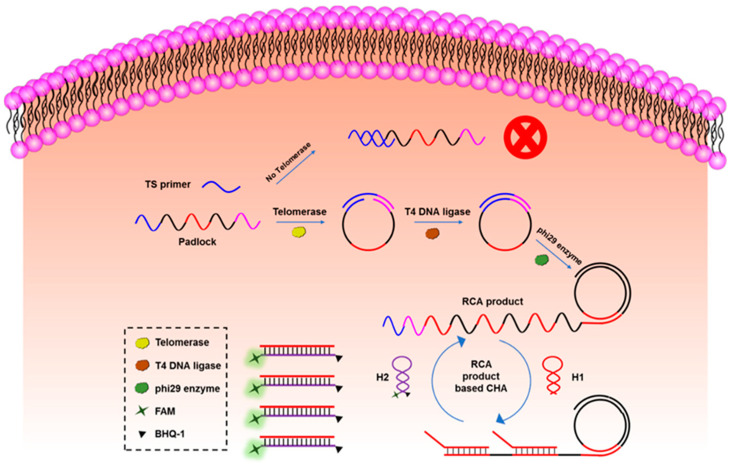
The principle of CHA-assisted RCA for telomerase activity detection. Reprinted (adapted) with permission from [123]. Copyright (2020) American Chemical Society.

The cyclic amplification techniques based on hairpins, which mainly include HCR and CHA, are developed to enhance the biosensing signal largely. HCR was triggered by an initial single strand of nucleic acid, and the DNA hairpins with sticky ends alternately hybridized to form a long double helix. Hou et al. developed an HCR electrochemical sensor based on the signal attenuated mode for detecting micro-RNA with high selectivity [121]. The sensor showed good sensitivity with a LOD of 1 pM. HCR may be triggered without the existence of the target and cannot be stopped automatically, while CHA needs the participation of original ss-DNA as the trigger in every reaction round, which makes the biosensors based on CHA more robust. Duan et al. proposed a fiber optic biosensor which was based on CHA and nanocomposites-assisted signal amplification to detect 18 samples of food [122]. The LOD of the sensor could reach 12 pM. In addition, researchers are trying to combine CHA with other advanced biomedical analysis methods. Wu et al. combined CHA with rolling circle amplification (RCA) for the telomerase activity detection with high sensitivity both in vitro and in situ [123]. As shown in Figure 6, the RCA step was used to monitor the activity of telomerase and amplify the target, and the CHA step was used to convert the increase of the RCA product into fluorescence enhancement to readout.

## 4. DNA Templated Biosensors

With the development of nanotechnology, DNA is being considered as an essential material that can manufacture various programmable structures in virtue of DNA hybridization’s high selectivity [124,125]. These structures can be used as the template to design more smart biosensors with high resolution and addressable anchors [47,126,127]. In this session, the applications of different DNA templates in biosensors, including DNA tiles and DNA origami, are introduced.

### 4.1. DNA Tile Assembly and Its Application

DNA tile was assembled by Seeman et al. in 1983 for the first time [128]. Using the ss-DNA assembly, they obtained the DNA lattice with the four-arm junction called the “Holliday junction” [129,130] and represented their results by electrophoretic and UV optical absorbance methods. Based on this work, more complex 2D nanostructures like multi-arms junction [131,132], double crossover tile (DX tile) [133,134,135], crossover tile (TX tile) [136,137] and 4 × 4 cross tile [138] were proposed. Three-dimensional nanostructures could also be constructed by using DNA tiles. One method is to use DNA tiles with multiple sticky ends as vertices of the nanostructure [139,140]. Another method is to use single-stranded DNA to form planes, which are stitched together into a three-dimensional structure later. This method can be used to construct many complex 3D structures, such as a DNA tetrahedron, heteroprism, and biprism [141,142,143].

**Figure 7:**
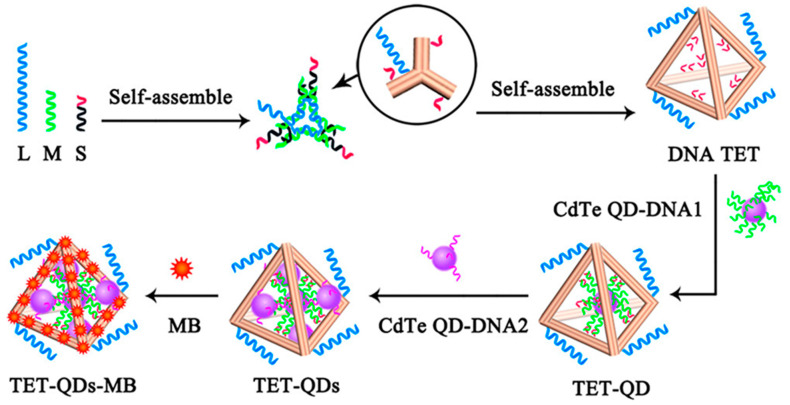
The DNA tetrahedron served as the immobilized template of CdTe QDs-Methylene Blue for photoelectrochemical detection. Reprinted (adapted) with permission from [155]. Copyright (2018) American Chemical Society.

Several DNA tiles, like DNA tetrahedron structures, have been successfully applied to construct biosensors due to their regulable size, relatively high rigidity, and multiple anchoring points. Different DNA tetrahedron sensors based on optical sensing [52,144,145], electrochemical sensing [146,147], SPR sensing [148,149,150], and intracellular imaging [151] were widely reported. By using a DNA tetrahedron-based template, the density regulation of bioprobes on the sensor surface could be optimized to enhance the biosensing performance [152,153,154]. Li et al. designed a kind of ultrasensitive photoelectrochemical (PEC) biosensor based on the DNA tetrahedron as the immobilized template for CdTe QDs-Methylene Blue (shown in Figure 7) [155]. Their proposed PEC biosensor had a linear range of 50 aM to 50 pM for miRNA-141, with a LOD of 17 aM. The DNA tetrahedron also has the stronger resistance to the enzyme attack to ensure a more durable lifetime in tissue and organism [156,157]. Furthermore, the DNA tetrahedron also has the ability to precisely control the fluorescence signal intensity by adjusting the size and natural rigidity of the tetrahedron structure, which is essential for the fluorescence biosensing methods in vivo [158]. Although DNA tiles can be widely used in biosensors for both in vitro and in vivo clinical diagnostics, the construction of more complex DNA tiles-based patterns requires a tedious predesign process and a high level of experimental operation, which hinders further application of DNA tiles in biosensors. That is, more robust, efficient, and universal DNA assembly technology is required.

### 4.2. DNA Origami Assembly and Its Application

Inspired by the DNA lattice assembly, Rothemund proposed the DNA origami methodology to construct confined patterns [159], which made it possible to construct complex nanostructures on the biosensor surface. The basic principle of DNA origami is to transform folded single-strand DNA (called “scaffold strands”) into well-defined patterns through the raster fill technique. By extending or functionalizing ssDNA in DNA nanostructures, target molecules (specific biological receptors, signal-reporting molecules, or functional materials used to enhance sensor sensitivity) can be precisely anchored to predesigned sites. Such permutation ability can be used to tune the distribution of biological probes on the biosensor surface and construct novel biosensors.

**Figure 8:**
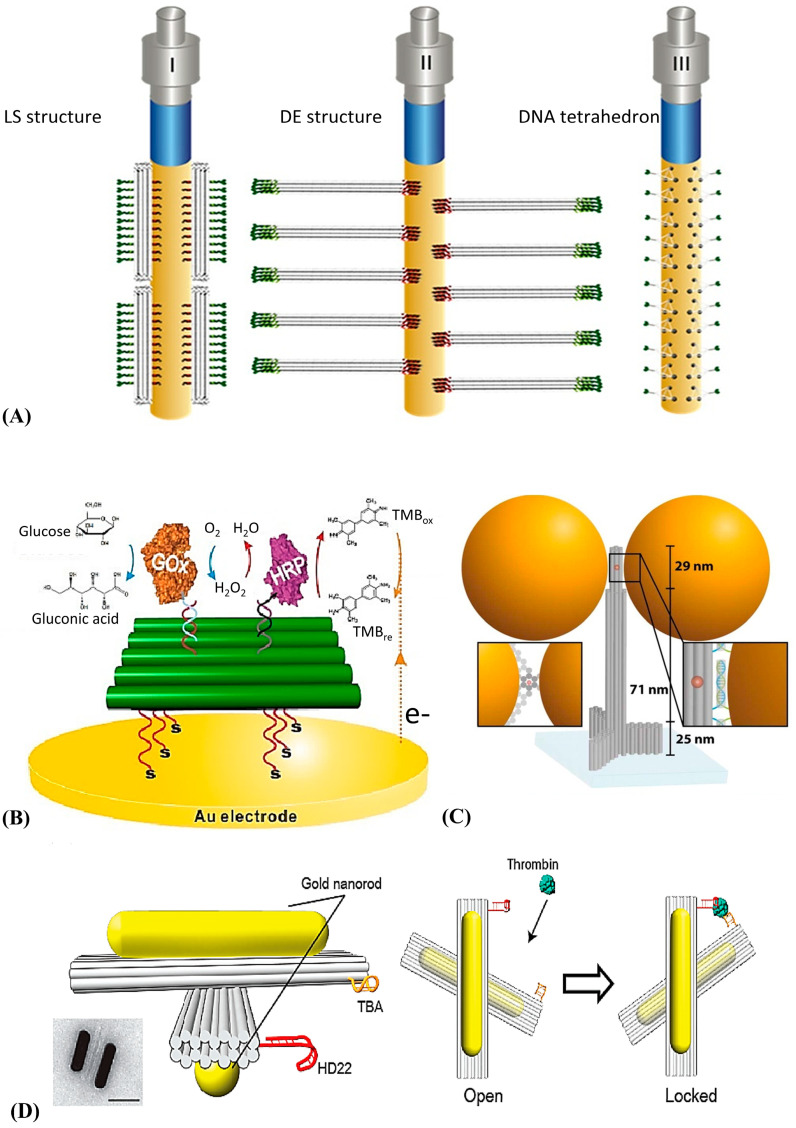
(**A**) The DNA origami LS (left), DE (middle), and DNA tetrahedrons (right) as the anchoring point of the thrombin-specific aptamers on the SPR biosensor. Reprinted (adapted) with permission from [160]. Copyright (2018) American Chemical Society. (**B**) The enzyme-catalyzed reactions are controlled by adjusting the distance between the GOx and HRP. Reprinted (adapted) with permission from [163]. Copyright (2019) American Chemical Society. (**C**) The DNA origami pillar was constructed to build the optical nanoantenna with two 100 nm Au nanoparticles together. Reprinted (adapted) with permission from [177]. Copyright (2021) American Chemical Society. (**D**) The schematic diagram of the reconfigurable 3D plasmonic metamolecules [182]. Copyright (2019) MDPI, Basel, Switzerland, under the Creative Commons Attribution License.

Generally, the bioprobe’s distribution could be controlled by adjusting the bioprobe’s concentration, shortening the incubation time, or adding backfilling molecules. However, the steric hindrance effect, caused by the bioprobe’s surface aggregation, would prevent the target molecules from combining with the probes. DNA origami could be a good candidate. Daems et al. immobilized a 24-helix bundle origami on the surface of the SPR sensor as the programmable anchoring point for aptamer probes [160] (Figure 8A). A more comprehensive detection range and better linearity could be realized. This might be attributed to the controlled or high density of multiple biological receptors in each nanostructure and their upward orientation. Moreover, their supposed method did not need to backfill the sensor surface after the modification process. Recently, they evaluated the facilitation of biomolecular interactions on the nanoscale biosensor interface constructed by DNA origami [161]. A fourfold increase in binding dynamics and a sixfold increase in binding efficiency compared with direct immobilization were realized. DNA templates with addressable sites also provided a suitable platform for enzyme-assisted biosensors [162], which could increase the local concentration of intermediate products and reduce interference from the other components. Then, the catalytic efficiency of the enzyme could be enhanced dramatically. Ge et al. constructed the sub-monolayer DNA origami scaffold on the sensor electrode to study the redox enzymatic cascade pathways [163]. Glucose oxidase (GOx) and horseradish peroxidase (HRP) were bound to DNA origami with DNA-protein conjugation and double-stranded hybridization (Figure 8B). With the decrease of distance between GOx and HRP from 65 to 10 nm by adjusting the anchoring sites, the enzyme activity increased dramatically.

Moreover, the DNA template also shows great advantages in organizing function materials with well-defined spatial distributions [164,165,166,167,168], especially on complex surfaces [169,170,171,172,173]. One widespread application is the assembly of optical antennas for single-molecule detection [174]. The orderly arrangement of local plasmon structures on the surface of DNA origami can generate significant electric field enhancement at the nanometer gap, increasing the quantum yield of fluorescent molecules [175] or surface-enhanced Raman scattering [176]. Acuna et al. proposed a kind of self-assembled DNA origami-based optical nanoantenna for single-molecule detection [177] (Figure 8C). They used mercapto-modified oligonucleotides to construct a gold nanoparticles dimer with 29 nm gaps on the rod-like DNA origami. They used quenching agents to reduce the fluorophore’s inherent quantum yield to evaluate the self-assembled antennas’ fluorescence enhancement. The experimental results showed that the fluorescence enhancement index of this antenna reached 306, and it was not influenced by the type of fluorophore. Additionally, it was demonstrated that these antennas could be used to detect a single molecule with a concentration of 25 μM.

Furthermore, DNA has good dynamic characteristics because of its natural flexibility, which indicated that it was accessible to control the formation change of the DNA template through external stimuli [178], such as chain displacement reaction [179], adjusting the electric field [180], and introducing anchor points modified with azobenzene or other photosensitive groups [181]. These new approaches could be applied to construct novel nanodevices. An example widely reported was the combination of Au nanorods and Entropy-control conformational DNA origami, which could construct reconfigurable 3D plasmonic biosensors based on circular dichroism (CD) changes in the visible wavelength range [182]. As shown in Figure 8D, two Au nanorods were immobilized on the DNA origami dimer with two crossed DNA origami sticks that could rotate each other in the warp direction. The decussate Au nanorods excited different intensities of the CD spectrum as the angle between the nanorods changed [183]. By introducing the specific DNA aptamers to the thrombin on the origami sticks, the binding event between the thrombin and the DNA aptamer could be converted into a change in the origami conformation, which could finally read out as a change in the intensity of the CD spectrum. This method was taken for detecting thrombin with a LOD of 100 pM.

However, a prominent drawback of DNA origami is the contradiction between the requirements of the DNA origami with the larger scale (for example, constructing larger periodic patterned surfaces to integrate more functions into a single DNA origami structure), and the scarcity of the longer natural DNA chain suitable for the scaffold chain. A forthright way was to use longer synthetic ss-DNA as the scaffold strand [184,185]. However, due to the high cost of synthesis, it was very difficult to scale up. A more effective and practical strategy was combining the DNA tile assembly methods with DNA origami. Seeman et al. proposed a kind of crystalline 2D-DNA origami array with the edge dimension of 2–3 μm [186]. Yao et al. proposed a kind of six-helix bundle DNA origami (called “M-DNA”) to mimic the ss-DNA in nature [187]. In the study, they used the M-DNA structure to perform the strand-displacement reaction to demonstrate the dynamic feature of the M-DNA.

## 5. Summary and Conclusions

In this review, the basic principles and the recent advances of biosensors based on functional DNA strands, DNA hybridization, and DNA templates are summarized. Table 1 showed their advantages, disadvantages, and detection object.

Functional DNA strand-based biosensors mainly consist of DNA aptamer biosensors and DNAzyme biosensors. DNA aptamer is considered to be a powerful alternative to traditional bioprobes (like the enzyme and the antibody), because of its high stability, adjustable affinity, and selectivity to various biotargets. The DNAzyme-based biosensing methods set the detection targets as the trigger of the catalytic reaction. During the reaction time, the product of the reaction accumulates to increase the response of the sensor. Therefore, under the long period of incubating time, the biosensing strategy based on DNAzyme often shows higher sensitivity.

DNA hybridization-based biosensors consist of DNA hairpin biosensors, HCR biosensors, and CHA biosensors. The DNA hairpin is suitable for detecting nucleic acid fragments in electrochemical sensors or FRET sensors. Because the highly specific hybridization between the hairpin and the nucleic acid target causes a conformation change of the hairpin, it could be easily converted to an electrochemical activity or a fluorescence intensity change. HCR and CHA biosensors both use the detection target to stimulate the DNA hairpin amplification process. The difference between them is that the HCR cannot stop automatically once it is activated until the substrate DNA hairpin is depleted, while CHA requires the participation of the detection target in each round of the amplification. A noticeable advantage of CHA and HCR is that the substrate hairpin, the primary structure of which is physically undamaged, can be recyclable through certain annealing protocols.

DNA templated-based biosensors consist of DNA tile-based biosensors and DNA origami-based biosensors. DNA tile-based biosensors like the DNA tetrahedron biosensors could precisely adjust the surface density of the bioprobes in the biosensing interface, which is helpful for improving the capture efficiency of targets and bioprobes. DNA origami, which can offer a compositive biosensing platform for anchoring bioprobes and function materials with nanoscale accuracy, is helpful to adjust the dynamics on the biosensing interface and to develop advanced biosensors for single-molecule detection.

## 6. Future Perspective

Although DNA biosensors have great potential in many areas, there are still some problems, like the latent toxicity of the DNA biosensors, the stability of the DNA hybridization-based biosensors, the complexity to fabricate template-based DNA biosensors, and the difficulty to design certain DNA structures for different biosensors.

Firstly, the biotoxicity of the modified DNA on the sensor surface may influence the biosensor’s safety. For example, in vivo detection, DNA immobilized on the biosensors may over-induce the immune response of people. Therefore, it is necessary to find robust and biocompatible sensor packaging methods to avoid direct contact between the modified sensor surface and the human tissue. Nontoxic hydrogel can be used potentially to wrap the vivo DNA biosensor to prevent the modified DNA from directly contacting with people.

Secondly, the stabilization of the spatial structure of DNA assembly needs to be improved. The fluctuation of the outside environment may result in the adverse conformation change of the multimer formed by the DNA hybridization, which may cause the invalidation of the biosensors. For example, in HCR, spontaneous trigger assembly reactions of DNA hairpins may be nonspecifically triggered by overly high temperature. Therefore, it is necessary to improve the tolerance of DNA biosensors to a wider range of temperatures. Adjusting the ratio of different nucleic acids and inserting the manual modified nucleic acids into the DNA sequence to increase its melting temperature seem to be efficient strategies.

Thirdly, the fabrication process of the complex DNA supermolecule is still burdensome. For example, the present assembly process based on the one-pot annealing protocol requires a slow cooling rate, which is time-consuming. To solve this issue, adjusting the assembly temperature and the annealing rate according to different DNA origami structures seems to be a possible way. Another feasible solution is to accelerate the annealing process by gradually replacing the component of the DNA template solution [188].

Lastly, the designing process of different DNA structures is experience-based, which limits the wide application of DNA among different biosensing fields. Thus, methods which could simplify the DNA designing process need to be proposed. A potential method is developing more automated DNA designing software to help researchers simplify the DNA structure designing process.

## Figures and Tables

**Table 1:** Summary of the different types of DNA-based biosensors.

Category		Advantages	Disadvantages	Detection Object
Functional DNA strand-based biosensors	DNA aptamer	Easily accessed; easily modified; adjustable affinity; more economic; more durable lifetime	Requires multi-round selection; easily attacked by the nucleic enzyme; potential biotoxicity	IFN-γ [64,65]
				Pb^2+^ [77]
				Thrombin [69,83]
	DNAzyme	High catalytic activity; small molecule detection with high sensitivity	Easily affected by temperature; needs oxidative substrate; the reaction product cannot be recycled to use	Pb^2+^ [102]
				AMP, Lyso [106]
				Bleomycin [107]
DNA hybridization-based biosensors	DNA hairpin	Detects nucleic acids with high selectivity; easily converts the hybridization process into physical signal change	Easily damaged by temperature	DNA [117,118,119]
	HCR	High sensitivity, especially at the biosensing interface	Easily be triggered automatically by mistake	micro-RNA [121]
	CHA	More stable than HCR	Not as sensitive as HCR	micro-RNA [122]
DNA template-based biosensors	DNA tiles	Effectively adjust the surface density of bioprobes; suitable for in vivo biosensing	Lack of the ability to form complex and large-scale patterns	mi-RNA 141 [155]
	DNA origami	Control the arrange of bioprobes and materials with nanoscale accuracy; programmable nanostructure	Time-consuming annealing process; expensive; difficult to design	Oligonucleotides [177]
				Thrombin [160,182]
